# An Almost Herbst’s Triad: Finger Clubbing and Iron Deficiency Anemia Associated With Severe Gastroesophageal Reflux

**DOI:** 10.1097/PG9.0000000000000234

**Published:** 2022-07-25

**Authors:** Paola Blagec, Mia Šalamon Janečić, Iva Hojsak

**Affiliations:** From the * Referral Center for Pediatric Gastroenterology and Nutrition, Children’s Hospital Zagreb, Zagreb, Croatia; † School of Medicine, University of Zagreb, Zagreb, Croatia; ‡ School of Medicine, University J. J. Strossmayer, Osijek, Croatia.

**Keywords:** anemia, children, esophagitis, finger clubbing, GERD

## Abstract

Digital clubbing is most commonly caused by pulmonary or cardiac disease, and only rarely it is a manifestation of a gastroenterological disorder. We report a pediatric patient in whom severe gastroesophageal reflux disease caused anemia and digital clubbing, partial clinical presentation of the Herbst triad.

## INTRODUCTION

Digital clubbing, enlargement of distal phalanges of the hands or feet (1), is most often associated with heart and lung disease, but it can sometimes be associated with some chronic gastrointestinal diseases. We present a patient who developed clubbing of the fingers because of erosive esophagitis caused by gastroesophageal reflux disease (GERD).

## CASE REPORT

**Fig 1.**
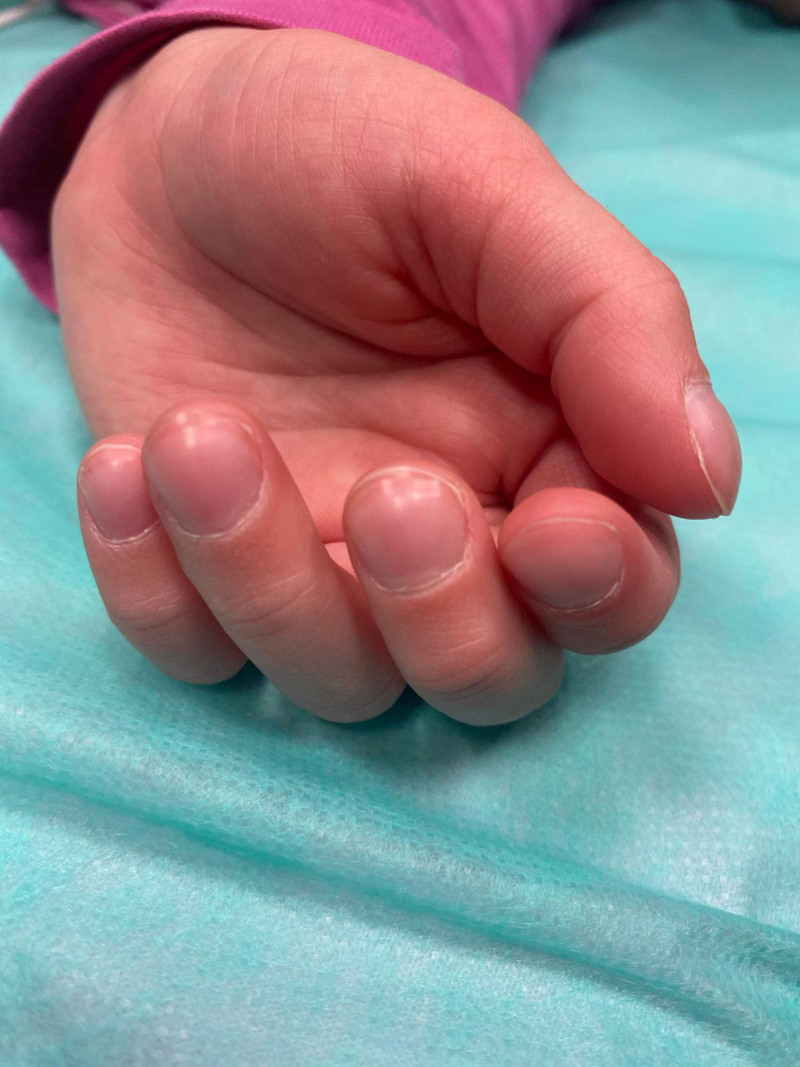
Left hand showing clubbing of the fingers.

**Fig 2.**
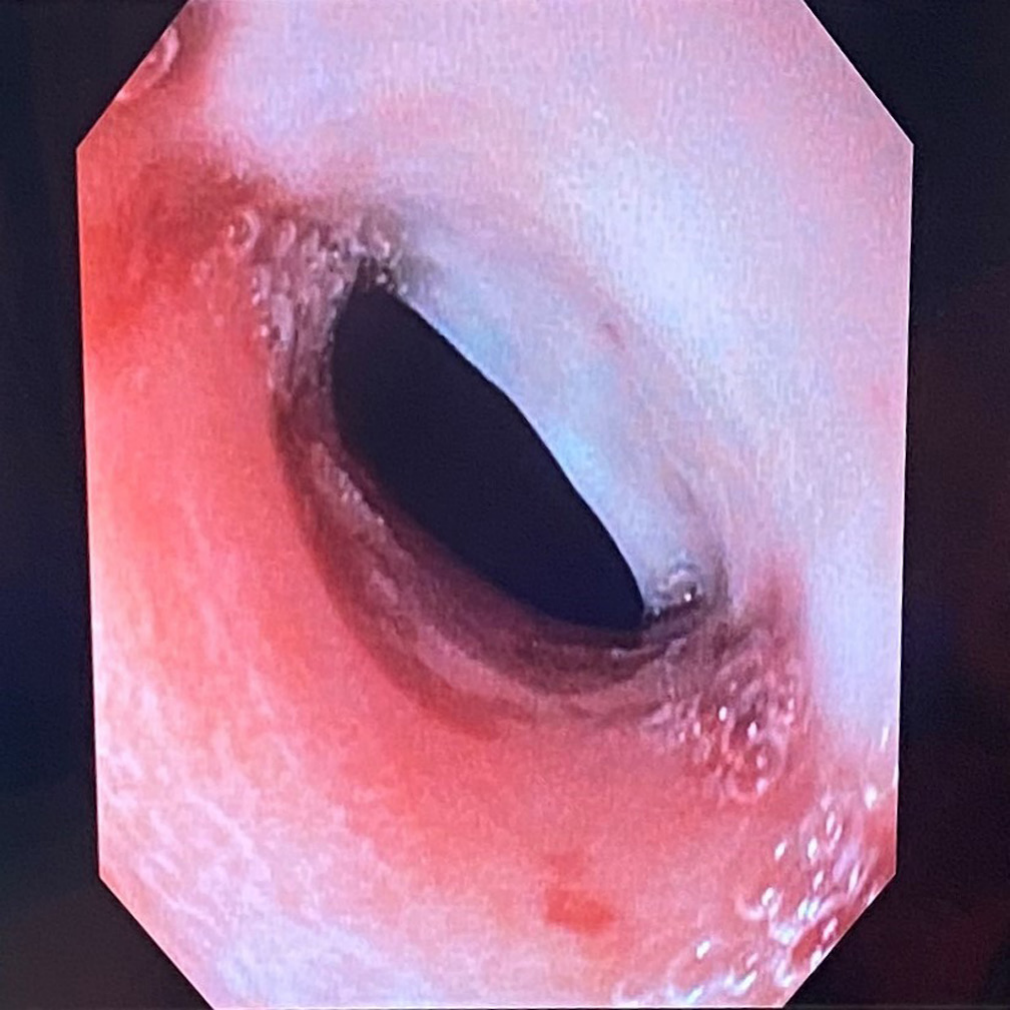
Endoscopy revealing mild stenosis of the esophagus.

A 6-year-old girl was admitted for further evaluation for 4 months of dysphagia, profuse salivation, abdominal pain, and anemia. She reported no other symptoms. Initial physical examination revealed clubbing of the fingers (Fig. 1) and toes and pale skin. She had no other signs of malnutrition body weight was 19 kg (Z score –0.98) and height 118 cm (Z score –0.21). Laboratory findings revealed microcytic anemia (Hb 74 g/L, MCV 57.0 fL), with low iron levels, normal liver enzyme levels, blood urea nitrogen, and creatinine level. Cardiology and pulmonary evaluations were normal. Gastroesophageal symptoms and anemia led to upper endoscopy that showed hyperemic esophageal mucosa with extensive erosions and fibrin deposits (esophagitis grade D per Los Angeles classification), a mild stenosis 22 cm from the tooth ridge that was passed by an adult endoscope, and a minor hiatal hernia (Fig. 2). Histology revealed inflammatory changes in the upper third of the esophagus and cardiac metaplasia of the mucosa with inflammatory infiltration of plasma cells and eosinophils (20–30 eosinophils per high-power field) on multiple biopsies from the middle and lower third of the esophagus, but no intestinal metaplasia or dysplasia. Multichannel intraluminal impedance-pH monitoring confirmed an elevated number of reflux episodes, with a total of 368 reflux episodes registered on impedance; 121 were acidic, 221 weakly acidic, and 26 nonacidic. The 24-hour pH monitoring showed 93 acidic episodes, the longest lasting 11.2 minutes, and the reflux index was 6.5% (upper limit of normal). All other diagnostic tests (x-ray of the thorax and abdomen, abdominal ultrasound, barium swallow, serologic screening for celiac disease, cytological analysis of peripheral blood smears, hemoglobin electrophoresis, electrocardiogram, and heart ultrasound) were normal. She was treated with a proton pump inhibitor (PPI–esomeprazole 40 mg once daily, 2 mg/kg/day), oral iron, and antireflux measures. The treatment reduced swallowing difficulties. A follow-up endoscopy performed 2 months later revealed significant improvement; while signs of inflammation were still present, no erosions or stenosis was seen. Esophageal cardiac metaplasia remained. Complete blood count was normal. As discomfort was reduced and endoscopic findings had improved, an attempt was made to reduce the PPI dosage, but after dose reduction, symptoms worsened. Therefore, the patient underwent Nissen fundoplication that decreased the symptoms.

## DISCUSSION

We present a rare manifestation of finger clubbing likely caused by erosive esophagitis due to GERD. Digital clubbing is usually a manifestation of chronic pulmonary or cardiac disease. It is sometimes associated with chronic gastrointestinal disease such as inflammatory bowel disease, celiac disease or cirrhosis, but GERD as a cause is rarely reported (3-6). Previous reports include younger children or children with neurological impairment. Our 6-year-old girl has normal development but manifested digital clubbing before significant symptoms of GERD appeared.

The pathophysiological mechanisms of clubbing remain unclear. Some histological findings show that clubbing is a result of vascular changes of the smallest vessels in fingertips, although these findings are inconsistent (7). However, it is hypothesized that vascular endothelial growth factor (VEGF) plays a key role in local changes. A few mechanisms that lead to increased local VEGF production are proposed: chronic inflammation, circulatory disorders, and neurocirculatory mechanism (1,5).

The Herbst triad is defined by the presence of GERD associated with digital clubbing, anemia, and hypoalbuminemia caused by protein–losing enteropathy (3). Indeed, the majority of reported cases had Herbst’s triad (digital clubbing, anemia, and GERD) also low albumin levels. In the last published case, albumin levels were not mentioned (6). Our patient did not have hypoalbuminemia. We speculate that the reason may be in the duration of symptoms; our patient, a normally developed 6-year-old girl, could easily communicate her symptoms before hypoalbuminemia developed.

Herein, we report an unusual manifestation of a recognized disorder. Providers must remember that gastrointestinal disease can also be associated with digital clubbing. Considering gastrointestinal disorders when clubbing is present is especially important in patients who cannot communicate their symptoms, young patients, and patients with severe neurological impairment.

## ACKNOWLEDGMENTS

M.Š.J. and I.H. contributed to the patient treatment. P.B., M.Š.J., and I.H. prepared the article. All authors read and approved the final version of the article.
